# High serum uric acid levels are associated with increased prevalence of gallstones in adult women: a cross-sectional study based on NHANES

**DOI:** 10.3389/fmed.2025.1487974

**Published:** 2025-01-17

**Authors:** Guozheng Lv, Decai Wang, Yu Huang, Ruizi Shi, Chuan Qin, Xi Chen, Xintao Zeng, Hua Luo, Pei Yang, Sirui Chen, Jianjun Wang

**Affiliations:** ^1^Department of Hepatobiliary Surgery, School of Medicine, Mianyang Central Hospital, University of Electronic Science and Technology of China, Mianyang, China; ^2^Tongji Medical College, Huazhong University of Science and Technology, Wuhan, China; ^3^Department of Urology, School of Medicine, Mianyang Central Hospital, University of Electronic Science and Technology of China, Mianyang, China; ^4^NHC Key Laboratory of Nuclear Technology Medical Transformation, School of Medicine, Mianyang Central Hospital, University of Electronic Science and Technology of China, Mianyang, China

**Keywords:** gallstones, serum uric acid, NHANES, women, cross-sectional study

## Abstract

**Objective:**

We investigated the association between serum uric acid (SUA) levels and gallstone (GS) prevalence in adult women.

**Methods:**

Participants' information were taken from the United States National Health and Nutrition Examination Survey (2017–2020). Logistic regression analysis and dose-response curve were used to assess the association between SUA levels and the prevalence of GS in adult women. Subgroup analyses were performed to investigate associations between SUA levels and age, ethnicity, body mass index, hypertension, and diabetes.

**Results:**

A total of 600 participants self-reported a history of GS. After adjusting for confounding, the prevalence of GS in adult women increased by 14% for every 1 mg/dL increase in SUA (odds ratio [OR]: 1.14, 95% confidence interval [CI]: 1.06, 1.22). Testing SUA as a categorical variable for sensitivity analyses indicated a 1.6-fold increase in the prevalence of GS in tertile 3 (OR=1.60, 95% CI: 1.25, 2.04) compared to tertile 1. Dose-response curves showed a nonlinear correlation between SUA levels and the prevalence of GS. Subgroup analyses indicated that SUA level was associated with an increased prevalence of GS in most subgroups, although subtle differences existed.

**Conclusion:**

SUA was positively and non-linearly associated with the prevalence of GS in adult females. Despite the inability to clarify the causal relationship between them, our results remain interesting.

## 1 Introduction

Gallstones (GS) are a common disease associated with the digestive system, and their prevalence is increasing worldwide (1). Approximately 800,000 of the adult population in the United States is affected by GS (2). Although benign, GS is also a high-risk factor for gallbladder cancer (3). In the population with GS, most patients are asymptomatic and are often incidentally diagnosed; a few patients may experience abdominal pain, nausea, vomiting, fever, and other discomforts (4). In addition, patients may experience cholangitis, pancreatitis, and even infectious shock when stones enter the common bile duct, which can be life-threatening in severe cases (4). Therefore, most patients undergo surgery. According to the composition, GS are classified into cholesterol, melanin, and mixed stones (5), of which cholesterol stones account for more than 80% (6).

Epidemiological investigations have shown that the prevalence of GS is generally higher in women than in men (7–9). Several factors influence this difference. For example, higher estrogen levels in women increase cholesterol secretion through several pathways, leading to an increase in the concentration of cholesterol in bile, which increases the risk of gallstone formation (10–12). The gallbladder's lower responsiveness to cholecystokinin in women, combined with estrogen's impact on gallbladder smooth muscle function, leads to incomplete emptying and prolonged bile retention, increasing the likelihood of stone formation (13, 14). Genetic factors and lifestyle habits significantly influence GS development in women, with mutations in genes like *ABCG5/ABCG8*, more prevalent in women, increasing GS risk (10). A growing number of recent studies have pointed out that obesity and metabolic syndrome (MetS) are strongly associated with the pathogenesis of GS and that the prevalence of obesity and MetS is often higher in women than in men (15–17).

Serum uric acid (SUA) metabolism involves multiple steps and organs and is the end product of purine metabolism (18). Moderate levels of SUA are considered beneficial antioxidants; however, excessive SUA levels have been identified as an independent risk factor for a variety of diseases, including MetS, diabetes, hypertension, cardiovascular events, and kidney disease (19–21). However, the relationship between SUA levels and GS in women has yet to be effectively explored. We conducted this nationally representative cross-sectional study to deepen our understanding of the SUA-metabolism-related mechanisms of GS formation and help identify groups at high risk for GS.

## 2 Methods

### 2.1 Data source

**Figure 1:**
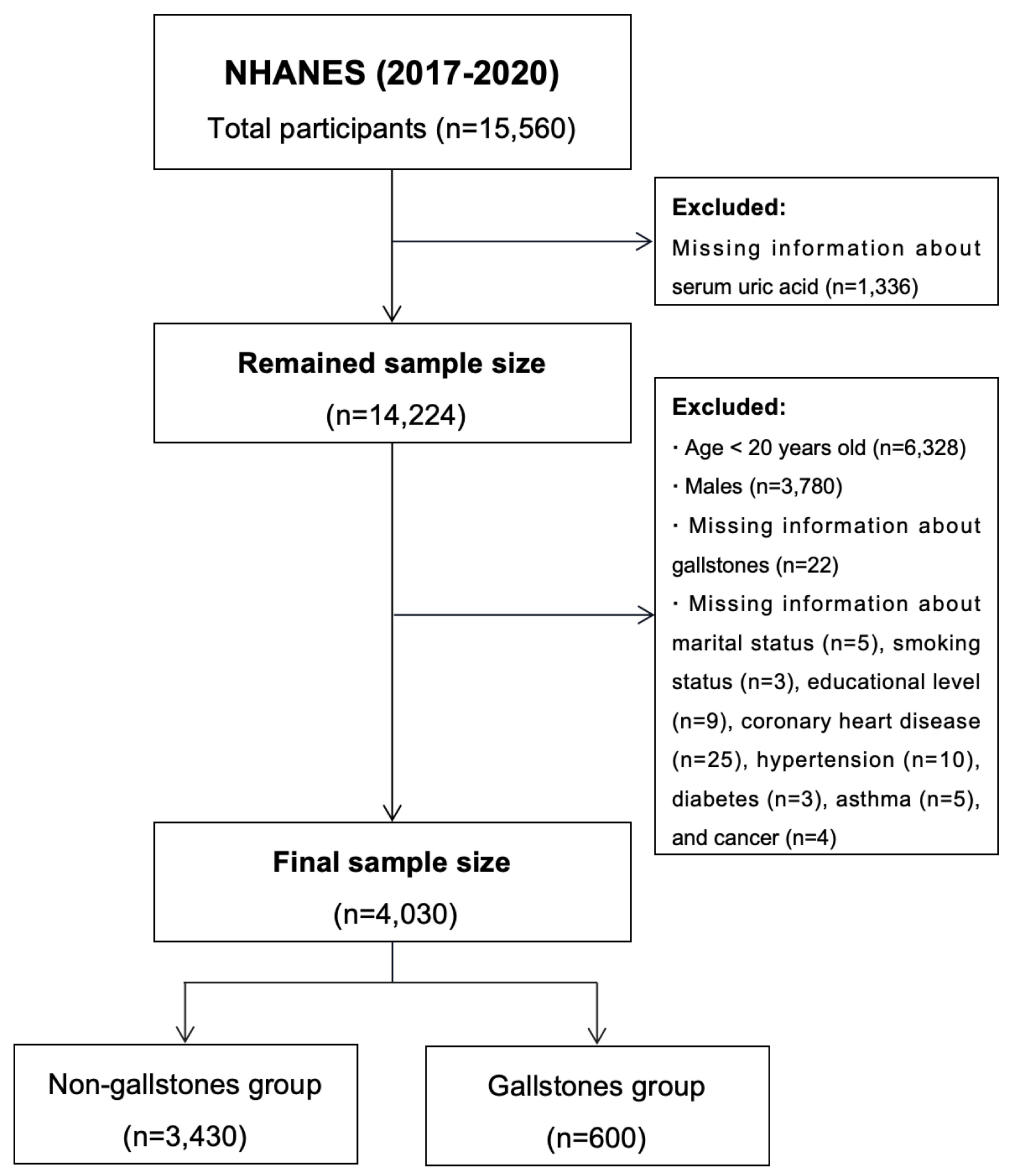
Flowchart for participants from NHANES (2017–2020). NHANES, National Health and Nutrition Examination Survey; SUA, serum uric acid; GS, gallstone; CHD, coronary heart disease.

The data of all study participants were extracted from the National Health and Nutrition Examination Survey (NHANES, https://www.cdc.gov/nchs/nhanes/index.htm.), a major survey conducted by the National Center for Health Statistics (NCHS). NHANES uses a stratified multistage probability sampling methodology (mainly consisting of the following steps: stratification, primary sampling units, secondary sampling units, household sampling, oversampling) to ensure that the survey sample is nationally representative. The participants encompassed diverse age, sex, ethnicity, and socioeconomic status groups. Step-by-step instructions for finding the NHANES dataset are provided in the Supplementary material. The data provided by NHANES help identify health priorities, develop disease prevention strategies, and public health policy. The NHANES also plays a vital role in preventing chronic diseases, improving nutrition, and promoting healthy lifestyles. The NHANES is updated every 2 years with a population of approximately 10,000 at a time and paused in March 2020 due to the COVID-19 epidemic. Questionnaires regarding GS and surgery were only used in the 2017–2020 cycle; participants from that cycle were selected for our study (Figure 1). All NHANES protocols were conducted in accordance with the U.S. Department of Health and Human Services Policy for the Protection of Human Research Subjects and are reviewed and standardized annually by the NCHS Research Ethics Review Board. All participants signed informed consent forms; therefore, there were no additional requirements for informed consent or ethical review for this study.

### 2.2 Data collection

The questionnaire was administered to adults aged ≥20 years. Patients with and without GS were assessed based on questionnaire results. The other covariates included in this study consisted of five main components: demographic variables, comorbidities, dietary intake factors, laboratory test results, and other parameters (Supplementary Table 1). Dietary data were collected using two 24-hour dietary recalls administered to each participant. The average intake from these two recalls was calculated and used in our analysis to ensure accuracy and account for daily variability. The procedures or methods of measurement for all covariates are available at www.cdc.gov/nchs/nhanes. SUA was the exposure variable, and GS prevalence was the outcome variable in this study.

### 2.3 Statistical analysis

Categorical variables are expressed as numbers (%). Continuous variables are expressed as mean ± standard deviation for normally distributed data and as medians and interquartile ranges for skewed distributions. Pearson's χ2 test and Fisher's test were used for comparisons of categorical variables, and the *t*-test or one-way analysis of variance was used for comparisons of continuous variables. According to these guidelines, we constructed multivariable logistic regression models to test the association between SUA levels, the prevalence of GS, and age at first gallstone surgery in adult women. Model 1: Covariates were not adjusted. Model 2: Adjusted for age, ethnicity, and marital status. Model 3 was adjusted for all potential confounders based on Model 2. Generalized additive model regression and smoothed curve fitting (penalized spline method) were used to assess the relationship between SUA levels and GS prevalence in adult women. A natural ratio test was used to calculate the inflection point when a nonlinear relationship was observed. Subgroup analyses were also performed for age, race, body mass index (BMI), and the presence of hypertension and diabetes. These subgroups were chosen due to their established or potential roles as modifiers or confounders in the relationship between serum uric acid levels and gallstone prevalence. For example, age and BMI are well-recognized risk factors for gallstones, while hypertension and diabetes are commonly associated with metabolic syndrome, which may share a pathophysiological link with serum uric acid levels and gallstone formation. All analyses were performed using R version 4.0.2 (http://www.R-project.org; The R Foundation) and Empower (www.empowerstats.com; X&Y Solutions Inc., Boston, MA, USA). Statistical significance was set at *P* < 0.05.

## 3 Results

### 3.1 Baseline characteristics

Finally, 4,030 participants were included in this study, including 600 in the GS group and 3,430 in the non-GS group. Compared with the non-GS group, SUA levels were significantly higher in the GS group [4.6 (3.9–5.5) vs. 5.1 (4.3–6.0), *P* < 0.001]. In addition, patients in the GS group were older and had higher odds of having hypertension, diabetes, asthma, coronary heart disease, and cancer (All *P* < 0.001). In addition, there were significant differences in BMI, ethnicity, physical activity level, marital status, alcohol consumption, education level, smoking status, poverty income ratio, total kcal intake, and triglyceride, high-density lipoprotein cholesterol, alanine aminotransferase, hemoglobin A1c, and ferritin levels between the two groups. More details are shown in Table 1.

**Table 1:** Baseline characteristics of NHANES participants (2017–2020).

**Characteristic**	**All (*n* = 4,030)**	**Non-GS group (*n* = 3,430)**	**GS group (*n* = 600)**	***P*-value**
SUA (mg/dL)	4.7 (3.9–5.6)	4.6 (3.9–5.5)	5.1 (4.3–6.0)	< 0.001
Age (years)	51 (35–64)	49 (34–63)	58 (44–70)	< 0.001
BMI	29.2 (24.7–34.8)	28.7 (24.3–33.9)	33.1 (28.0–39.2)	< 0.001
Ethnicity (%)				< 0.001
Non-Hispanic White	1,378 (34.19)	1,122 (32.71)	256 (42.67)	
Non-Hispanic Black	1,047 (25.98)	928 (27.06)	119 (19.83)	
Mexican American	923 (22.90)	766 (22.33)	157 (26.17)	
Other Race	682 (16.92)	614 (17.90)	68 (11.33)	
Physical Activity (%)				0.04
Never	1,424 (35.33)	1,187 (34.61)	237 (39.50)	
Moderate	1,469 (36.45)	1,257 (36.65)	212 (35.33)	
Vigorous	1,137 (28.21)	986 (28.75)	151 (25.17)	
Marital status (%)				< 0.001
Cohabitation	2,144 (53.20)	1,818 (53.00)	326 (54.33)	
Solitude	1,112 (27.59)	916 (26.71)	196 (32.67)	
Never married	774 (19.21)	696 (20.29)	78 (13.00)	
Alcohol consumption (%)				< 0.001
Never	456 (11.32)	394 (11.49)	62 (10.33)	
Ever	744 (18.46)	587 (17.11)	157 (26.17)	
Now	2,542 (63.08)	2,201 (64.17)	341 (56.83)	
Unclear	288 (7.15)	248 (7.23)	40 (6.67)	
Education level				0.02
Less than high school	708 (17.57)	591 (17.23)	117 (19.50)	
High school	920 (22.83)	763 (22.24)	157 (26.17)	
More than high school	2,402 (59.60)	2,076 (60.52)	326 (54.33)	
Hypertension (%)				< 0.001
No	2,552 (63.33)	2,255 (65.74)	297 (49.50)	
Yes	1,478 (36.67)	1,175 (34.26)	303 (50.50)	
Diabetes (%)				< 0.001
No	3,492 (86.65)	3,031 (88.37)	461 (76.83)	
Yes	538 (13.35)	399 (11.63)	139 (23.17)	
Asthma (%)				< 0.001
No	3,314 (82.23)	2,851 (83.12)	463 (77.17)	
Yes	716 (17.77)	579 (16.88)	137 (22.83)	
CHD (%)				< 0.001
No	3,934 (97.62)	3,364 (98.08)	570 (95.00)	
Yes	96 (2.38)	66 (1.92)	30 (5.00)	
Cancers (%)				< 0.001
No	3,601 (89.35)	3,105 (90.52)	496 (82.67)	
Yes	429 (10.65)	325 (9.48)	104 (17.33)	
Smoking status (%)				< 0.001
Never	2,708 (67.20)	2,352 (68.57)	356 (59.33)	
Ever	724 (17.97)	577 (16.82)	147 (24.50)	
Now	598 (14.84)	501 (14.61)	97 (16.17)	
PIR				0.04
< 1.3	1,057 (26.23)	905 (26.38)	152 (25.33)	
≥1.3– < 3.5	1,353 (33.57)	1,123 (32.74)	230 (38.33)	
≥3.5	1,086 (26.95)	935 (27.26)	151 (25.17)	
Unclear	534 (13.25)	467 (13.62)	67 (11.17)	
Total sugar intake	82.45 (51.12–123.29)	82.44 (50.61–123.30)	82.52 (54.01–122.05)	0.62
Total Kcal intake	1,731 (1,271–2,272)	1,740 (1,275–2,291)	1,652 (1,242–2,163)	0.01
Total fat intake	68.46 (47.59–96.67)	68.60 (47.62–97.24)	67.84 (46.79–91.36)	0.19
Total water intake	1,920 (840–3420)	1,980 (870–3,414)	1,920 (660–3,480)	0.29
TG (mmol/L)	1.22 (0.87–1.73)	1.19 (0.85–1.68)	1.45 (1.03–1.93)	< 0.001
TC (mmol/L)	4.81 (4.16–5.53)	4.81 (4.16–5.53)	4.81 (4.19–5.55)	0.98
HDL-C (mmol/L)	1.45 (1.22–1.73)	1.45 (1.22–1.73)	1.39 (1.16–1.60)	< 0.001
LDL-C (mmol/L)	2.79 (2.22–3.41)	2.79 (2.22–3.39)	2.79 (2.20–3.47)	0.99
ALT (U/L)	15 (11–21)	15 (11–20)	15 (12–23)	< 0.001
AST (U/L)	18 (15–21)	18 (15–21)	18 (15–22)	0.38
HbA1c (%)	5.6 (5.3–5.9)	5.5 (5.3–5.9)	5.7 (5.4–6.2)	< 0.001
Ferritin (ng/ml)	68.0 (32.7–129.0)	65.2 (31.7–124)	86.2 (40.4–159.5)	< 0.001

SUA, serum uric acid; BMI, body mass index; CHD, coronary heart disease; PIR, poverty income ratio; TG, triglyceride; TC, total cholesterol; HDL-C, high-density lipoprotein cholesterol; LDL-C, low-density lipoprotein cholesterol; ALT, alanine aminotransferase; AST, aspartate aminotransferase; HbA1c, hemoglobin A1c.

### 3.2 Logistic regression results between SUA and the prevalence of GS in adult women

Logistic regression analysis revealed a positive association between SUA levels and the prevalence of GS in adult women (Table 2). Even after adjusting for all potential confounders, this positive association persisted (odds ratio [OR] = 1.14, 95% confidence interval [CI]: 1.06, 1.22). Specifically, for every 1 mg/dL increase in SUA, the prevalence of GS in adult women increases by 14%. When we converted SUA from a continuous variable to a categorical variable (tertiles) for sensitivity analyses, we found a 1.6-fold increase in the prevalence of GS in adult women in tertile 3 (OR = 1.60, 95% CI: 1.25, 2.04) compared with SUA tertile 1.

**Table 2:** Results of logistic regression analyses of SUA and the prevalence of GS in adult women.

**Characteristic**	**Model 1 OR (95%CI)**	**Model 2 OR (95%CI)**	**Model 3 OR (95%CI)**
SUA	1.27 (1.20–1.35)	1.22 (1.14–1.30)	1.14 (1.06–1.22)
**Categories**			
Tertile 1	1	1	1
Tertile 2	1.46 (1.15–1.84)	1.38 (1.09–1.75)	1.32 (1.03–1.68)
Tertile 3	2.23 (1.78–2.78)	1.92 (1.52–2.43)	1.60 (1.25–2.04)

Model 1 = no covariates were adjusted.

Model 2 = Model 1 + age, ethnicity, and marital status were adjusted.

Model 3 = Model 2 + additionally adjusted education level, physical activity, smoking status, alcohol consumption, diabetes, hypertension, asthma, cancers, coronary heart disease, total cholesterol, triglyceride, high-density lipoprotein cholesterol, low-density lipoprotein cholesterol, alanine aminotransferase, aspartate aminotransferase, hemoglobin A1c, ferritin, poverty income ratio, total kcal intake, total fat intake, and total sugar intake.

### 3.3 Dose-response and threshold effects of SUA on the prevalence of GS in adult women

**Figure 2:**
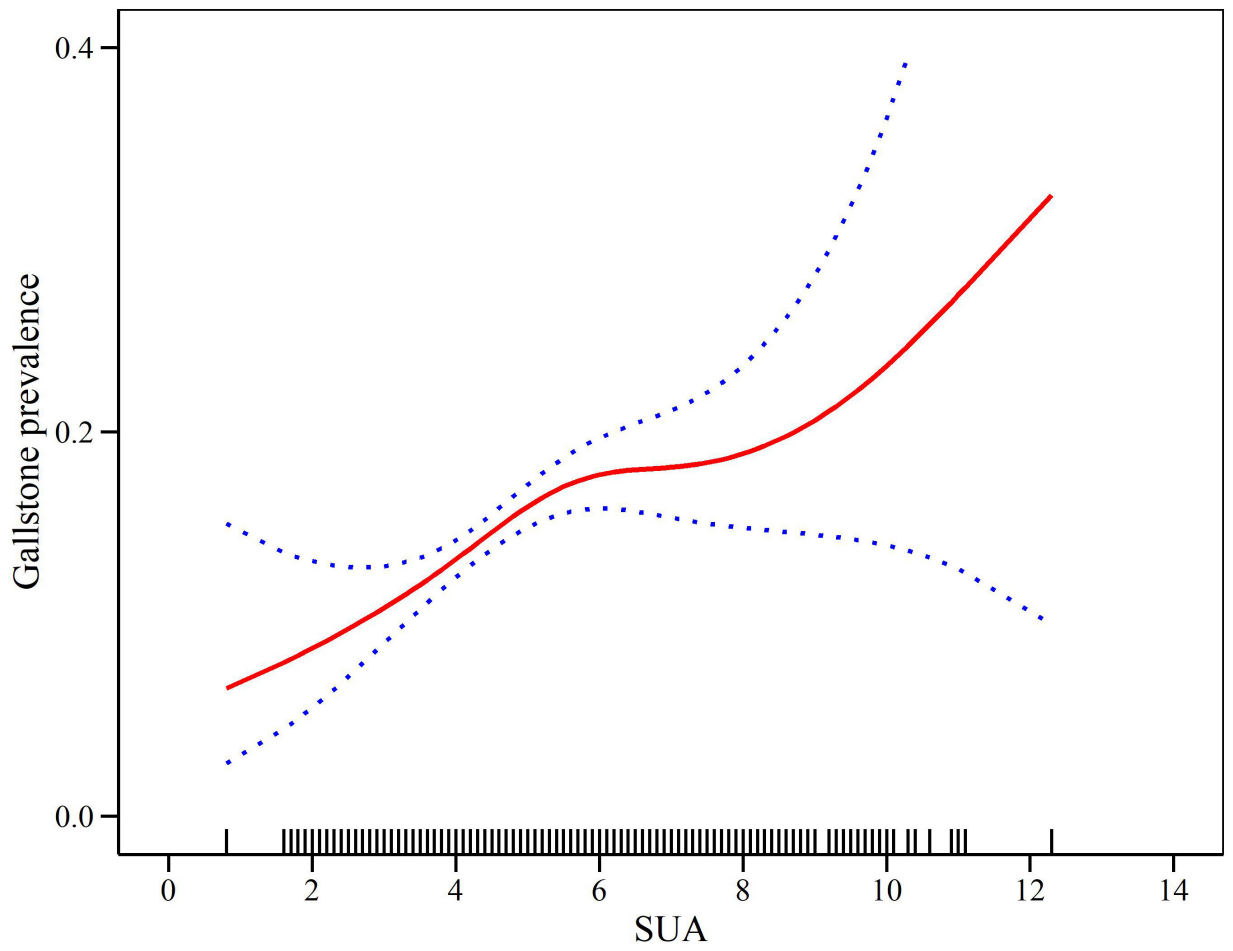
Density dose-response relationship between SUA with GS prevalence in adult women. The area between the upper and lower dashed lines is represented as 95% CI.

Additive generalized models and smoothed curve fitting were used to further explore the relationship between SUA levels and GS prevalence in adult women. Figure 2 and Table 3 show a nonlinear correlation between SUA levels and the prevalence of GS in adult women. Considering the effect of the saturation thresholds between them, the likelihood natural ratio test found that the best SUA threshold was 5.

**Table 3:** Two-piecewise linear regression and logarithmic likelihood ratio test explained the threshold effect analysis of SUA with GS prevalence in adult women.

**SUA**	**ULR test**	**PLR test**	**LRT test**
	**OR (95%CI)**	**OR (95%CI)**	***P*** **-value**
< 5	1.14 (1.06–1.22)	1.33 (1.13–1.56)	0.04
≥5				1.05 (0.94–1.17)	

ULR, univariate linear regression; PLR, piecewise linear regression; LRT, logarithmic likelihood ratio test, statistically significant: P < 0.05.

### 3.4 Subgroup analysis

Subgroup analyses further assessed the robustness of the relationship between SUA levels and GS prevalence in adult women across the subgroups. The results suggested that the prevalence of GS in adult women increased with increasing SUA levels, but there were subtle differences among the different subgroups (Table 4). Results indicated significant ORs in demographic groups: 1.24 (95% CI: 1.02, 1.50) for women aged 20–39, 1.35 (95% CI: 1.19, 1.54) for those aged 40–59, and 1.06 (95% CI: 0.96, 1.17) for individuals aged 60–80. Within different racial and ethnic groups, the ORs were as follows: 1.20 (95% CI: 1.08, 1.34) for non-Hispanic white individuals, 1.08 (95% CI: 0.94, 1.25) for non-Hispanic black individuals, 1.22 (95% CI: 1.04, 1.44) for Mexican-American individuals, and 0.99 (95% CI: 0.77, 1.27) for Other Race groups. Among the different BMI groups, ORs were 1.03 (95% CI: 0.81, 1.30) for ≤ 24.9 individuals, 1.12 (95% CI: 0.95, 1.33) for 25–29.9 individuals, and 1.08 (95% CI: 0.99, 1.18) for ≥30 individuals. The OR was 1.15 (95% CI: 1.05, 1.26) in those with hypertension compared with and 1.14 (95% CI: 1.02, 1.28) in the non-hypertensive population. The OR was 1.07 (95% CI: 0.93, 1.24) in those with diabetes and was 1.18 (95% CI: 1.08, 1.28) in the non-diabetes population.

**Table 4:** Subgroup analysis between SUA with GS prevalence in adult women.

**Characteristic**	**Model 1 OR (95%CI)**	**Model 2 OR (95%CI)**	**Model 3 OR (95%CI)**
**Stratified by age (years)**			
20–39	1.34 (1.13–1.58)	1.35 (1.14–1.60)	1.24 (1.02–1.50)
40–59	1.35 (1.21–1.51)	1.40 (1.24–1.58)	1.35 (1.19–1.54)
60–80	1.08 (0.99–1.18)	1.11 (1.02–1.22)	1.06 (0.96–1.17)
**Stratified by ethnicity**			
Non–Hispanic White populations	1.32 (1.20–1.45)	1.27 (1.15–1.40)	1.20 (1.08–1.34)
Non–Hispanic Black populations	1.22 (1.09–1.38)	1.16 (1.02–1.33)	1.08 (0.94–1.25)
Mexican American populations	1.38 (1.20–1.59)	1.28 (1.10–1.48)	1.22 (1.04–1.44)
Other Race	1.25 (1.01–1.54)	1.10 (0.88–1.37)	0.99 (0.77–1.27)
**Stratified by BMI**			
≤ 24.9	1.21 (0.99–1.48)	1.06 (0.86–1.32)	1.03 (0.81–1.30)
25–29.9	1.21 (1.06–1.39)	1.14 (0.98–1.33)	1.12 (0.95–1.33)
≥30	1.15 (1.06–1.25)	1.12 (1.02–1.22)	1.08 (0.99–1.18)
**Stratified by hypertension**			
No	1.28 (1.16–1.42)	1.20 (1.08–1.34)	1.14 (1.02–1.28)
Yes	1.16 (1.06–1.26)	1.19 (1.09–1.30)	1.15 (1.05–1.26)
**Stratified by diabetes**			
No	1.29 (1.20–1.39)	1.24 (1.15–1.34)	1.18 (1.08–1.28)
Yes	1.08 (0.96–1.22)	1.10 (0.96–1.25)	1.07 (0.93–1.24)

Model 1 = no covariates were adjusted.

Model 2 = Model 1 + age, ethnicity, and marital status were adjusted.

Model 3 = Model 2 + additionally adjusted education level, physical activity, smoking status, alcohol consumption, diabetes, hypertension, asthma, cancers, coronary heart disease, total cholesterol, triglyceride, high-density lipoprotein cholesterol, low-density lipoprotein cholesterol, alanine aminotransferase, aspartate aminotransferase, hemoglobin A1c, ferritin, poverty income ratio, total kcal intake, total fat intake, and total sugar intake.

### 3.5 Logistic regression results of SUA and age at first GS surgery in adult women

After adjusting for all potential confounders, the results showed no significant correlation between SUA and age at first GS surgery in adult women (β = −0.05, 95% CI: −0.88, 0.78) (Table 5).

**Table 5:** Results of logistic regression analyses of SUA and age at first gallstone surgery in adult women.

**Characteristic**	**Model 1β (95%CI)**	**Model 2β (95%CI)**	**Model 3β (95%CI)**
SUA	1.00 (0.15, 1.86)	0.98 (0.13, 1.83)	−0.05 (−0.88, 0.78)

Model 1 = no covariates were adjusted.

Model 2 = Model 1 + age, ethnicity, and marital status were adjusted.

Model 3 = Model 2 + additionally adjusted education level, physical activity, smoking status, alcohol consumption, diabetes, hypertension, asthma, cancers, coronary heart disease, total cholesterol, triglyceride, high-density lipoprotein cholesterol, low-density lipoprotein cholesterol, alanine aminotransferase, aspartate aminotransferase, hemoglobin A1c, ferritin, poverty income ratio, total kcal intake, total fat intake, and total sugar intake.

## 4 Discussion

This is the first study to investigate the relationship between SUA levels and the prevalence of GS in adult women. The results showed a nonlinear association between SUA levels and GS prevalence in a nationally representative sample of 4,030 adult women. More specifically, each 1 mg/dL increase in SUA level was associated with a 14% increase in the prevalence of GS in adult women. When SUA was converted from a continuous variable to a categorical variable, the prevalence of GS increased 1.6-fold in adult women in tertile 3 compared with those in tertile 1. The subgroup analyses showed that the positive association between SUA levels and the prevalence of GS in adult women remained in most subgroups. However, there were some differences between the subgroups.

The pathogenesis of GS is complex and has not yet been fully elucidated. Epidemiological surveys have shown that the prevalence of GS is higher in women than in men (7, 8). It is influenced by various physiological, hormonal, and lifestyle factors, the most important of which are sex hormone differences. Estrogens can promote cholesterol synthesis and secretion through a variety of pathways, including modulation of the classical “e2-esr1-srebp-2” pathway, up-regulation of *ABCG5/ABCG8* expression, and increased hepatic HMG-CoA reductase activity (10–12). Estrogen can also activate G protein-coupled receptor 30 through the signaling cascade of epidermal growth factor receptor, which inhibits cholesterol 7α-hydroxylase activity and bile acid synthesis, leading to an increase in cholesterol secretion and promoting gallstone formation (22). Pregnancy is associated with significantly higher progesterone levels, attenuating gallbladder motility and delaying gallbladder emptying, leading to cholestasis and stone formation (23). Additionally, the lipid metabolism in women differs from that in men. During the childbearing years, women store more fat and have a more active lipid metabolism, which can also lead to higher cholesterol levels and an increased risk of cholesterol stone formation (24–26).

Genetic factors also influence by genetic pathogenesis. Previous reports have shown that the prevalence of GS varies widely across ethnicities. The prevalence was higher in white populations than black populations (27, 28). Everhart et al. (28) found that the prevalence of GS in white women in the United States was 16.6% compared with 13.9% in black women, and the prevalence of GS in white men was 8.6% compared with 5.3% in black men. Our study also found a higher prevalence of GS in non-Hispanic white women than in non-Hispanic black women (32.71% vs. 27.06%, respectively). Such differences may be related to diet, lifestyle, and the level of healthcare among different races. Age is also an important risk factor for the development of GS (29, 30). The prevalence of GS increases significantly after age 40, with a 4- to 10-fold increase in GS prevalence in the older adult population (27). Our study also found that the GS group was significantly older than the non-GS group [58 (44–70) vs. 49 (34–63) *P* < 0.001]. As we age, the ability of the liver to synthesize and secrete cholesterol increases. In contrast, bile acid synthesis decreases, leading to higher cholesterol concentrations and a greater tendency to form cholesterol stones (31). In addition, the composition of bile changes with age. In older adults, the ratio of phospholipids to bile acids in bile decreases, cholesterol solubility decreases, and the risk of cholesterol crystal formation increases (27, 32). In addition, the motor function of the gallbladder decreases with age, leading to less efficient emptying of the gallbladder and cholestasis (27, 32). Emerging evidence suggests that gut microbiota may play a role in GS development by influencing bile acid metabolism, cholesterol absorption, and inflammation. Future research could explore the interplay between gut microbiota, metabolic factors, and SUA levels in GS pathogenesis to uncover novel therapeutic targets (9, 27, 33). Recent studies have also found that the incidence of GS is increasing at an alarming rate in younger populations and may be closely related to the prevalence of obesity, diabetes, MetS, and other diseases (33, 34).

SUA is the end product of purine metabolism and is closely associated with various metabolic diseases, including diabetes, hypertension, triglyceridemia, and MetS (35–39). Li et al. (18) found that for every 1 mg/dL increase in SUA level in a population with diabetes, there was an 8% and 5% increase in all-cause and cardiovascular disease mortality, respectively. Si et al. (38) found a significant positive correlation between SUA levels and the prevalence of triglyceridemia in US adolescents aged 12–18 years. Similarly, Che et al. (40) found that hyperuricemia was a risk factor for all-cause and cardiovascular mortality in people with hypertension and that asymptomatic hyperuricemia was significantly associated with a poor long-term prognosis. A recent study noted that SUA levels were negatively associated with lung function in the general population (41). In addition, SUA levels are strongly associated with the risk of developing MetS and related disorders in adolescents, and this relationship holds even in non-obese populations (42).

However, the mechanisms underlying the relationship between SUA levels and the GS remain unclear. Metabolic disorders may function as bridges between SUA and GS. SUA has an inherent antioxidant effect; however, at high levels, it can cause oxidative stress (43). SUA is capable of generating reactive oxygen species (ROS) via a peroxidase reaction, and excessive ROS can cause cellular damage, leading to lipid peroxidation, protein oxidation, and DNA damage (44, 45). Such oxidative stress can impair pancreatic islet cell function and reduce insulin secretion and activity, leading to insulin resistance (IR) (46, 47). IR is closely associated with the pathogenesis of GS pathogenesis (48). Next, SUA activates the NLRP3, a multi-protein complex that can activate the production of pro-inflammatory cytokines, such as IL-1β (49, 50), a potent inflammatory mediator that induces the release of other inflammatory factors, creating an inflammatory cascade response (50), which is closely associated with metabolic diseases (51). Oxidative stress can also induce adipocytes to secrete additional inflammatory factors, exacerbating metabolic disorders (52). SUA contributes to IR via various pathways. For example, SUA can affect insulin-mediated glucose uptake by inhibiting endothelial nitric oxide production, affecting vascular endothelium health (53). SUA can also inhibit key enzymes in the insulin signaling pathway, such as PI3K and AKT, through direct action on muscle and adipose tissues (44, 54). IR reduces glucose utilization, elevates blood glucose levels, and promotes excessive insulin secretion. A chronic high insulin state (hyperinsulinemia) increases adipogenesis, further exacerbating obesity and MetS (55, 56). The metabolic function of adipocytes is also affected by SUA, which activates the expression of specific genes, such as adipogenesis-related genes while inhibiting lipolytic enzyme activity and reducing lipolysis (57). These metabolic changes lead to fat accumulation in adipose tissue, increasing the risk of obesity. Obesity is a high-risk factor for the development of GS (58). Therefore, the mechanisms underlying the association between SUA levels and GS remain unclear. Future basic science research should focus on elucidating the precise molecular pathways, such as the role of oxidative stress, inflammation, and insulin resistance in linking SUA to GS formation.

Our findings suggest that elevated SUA levels may serve as a potential biomarker for GS risk in adult women. Clinically, SUA levels are routinely measured in patients, making them a practical and accessible marker for identifying individuals at higher risk for GS. Integrating SUA levels into risk prediction models for GS could improve early identification and prevention strategies. Furthermore, interventions aimed at lowering SUA levels through dietary, pharmacological, or lifestyle modifications might offer a novel approach to reducing GS risk, although further interventional studies are warranted to confirm these potential benefits.

Our study has several advantages. First, this is the first study to identify a relationship between SUA levels and the prevalence of GS in adult women. Specifically, SUA levels were positively and non-linearly associated with the prevalence of GS in adult women. Second, the NHANES encompasses a large number of participants, including people of different ages, sexes, races, and socioeconomic backgrounds, and the NHANES data are based on a national sample from the United States, which is highly representative. Our results provide a good representation of the health status of adult women in the United States. In addition, NHANES data were collected and processed by professionals following strict standards to ensure high quality and consistency. This standardization provided our findings with a high degree of reliability. Finally, we adjusted for potential confounders to ensure our findings apply to the general population.

Our study has some limitations. First, restricted by the nature of this cross-sectional study, we could not clarify the causal relationship between SUA levels and the prevalence of GS in adult women. Longitudinal cohort studies are necessary to clarify the temporal relationship and causative pathways. Second, the diagnosis of GS relied on participants' self-reports; thus, recall or reporting biases may exist. Third, the participants in this study were adult women aged ≥20 years; therefore, the applicability of our findings to men or populations < 20 years requires further investigation. In addition, our findings were based on adult women from the NHANES dataset. Additional studies are needed to validate these results in other populations, including men, younger individuals, and diverse ethnic groups, to enhance the generalizability of the findings. Furthermore, given the association between elevated SUA levels and GS, interventional studies could investigate whether lowering SUA levels through dietary, pharmacological, or lifestyle modifications could reduce the risk of GS. Moreover, NHANES only provides dietary data based on two 24-h recalls, which reflect short-term intake rather than long-term dietary habits. Thus, the data may not fully capture participants' habitual dietary patterns, which are crucial in GS pathogenesis. Finally, considering that SUA is associated with various metabolic disorders, future studies could explore the interactions between SUA, metabolic syndrome components, and GS in greater detail, including potential synergistic effects.

## 5 Conclusion

This study revealed, for the first time, the relationship between SUA levels and the prevalence of GS in women. Higher SUA levels are associated with an increased prevalence of GS in adult females.

## Data Availability

The original contributions presented in the study are included in the article/Supplementary material, further inquiries can be directed to the corresponding authors.
