# Supplementary material for: High serum uric acid levels are associated with increased prevalence of gallstones in adult women: a cross-sectional study based on NHANES
Source: Front Med (Lausanne). 2025 Jan 17;12:1487974. doi: 10.3389/fmed.2025.1487974 (PMC11782260; doi:10.3389/fmed.2025.1487974)
Supplement: Supplementary file 1 [file Table_1.docx]

**Supplementary Table 1. Covariates extracted from NHANES (2017-2020).**

| **Items** | **Composition** |
| --- | --- |
| Demographic variables | Age, Ethnicity |
| Comorbidities | Hypertension, Diabetes, Asthma, CHD, Cancer |
| Laboratory Results | SUA, TG, TC, HDL-C, LDL-C, ALT, AST, HbA1c, Ferritin |
| Dietary intake factors | Total Sugar, Total Kcal, Total Fat, Total Water |
| Others | BMI, Physical Activity, Marital Status, Alcohol consumption, Education Level, Smoking status, PIR |

NHANES, National Health and Nutrition Examination Survey; CHD, coronary heart disease; SUA, serum uric acid; TG, triglyceride; TC, total cholesterol; HDL-C, high-density lipoprotein cholesterol; LDL-C, low-density lipoprotein cholesterol; ALT, alanine aminotransferase; AST, aspartate aminotransferase; HbA1c, haemoglobin A1c; BMI, body mass index; PIR, poverty income ratio.
