# Supplementary material for: High serum uric acid levels are associated with increased prevalence of gallstones in adult women: a cross-sectional study based on NHANES
Source: Front Med (Lausanne). 2025 Jan 17;12:1487974. doi: 10.3389/fmed.2025.1487974 (PMC11782260; doi:10.3389/fmed.2025.1487974)
Supplement: Supplementary file 2 [file Data_Sheet_1.docx]

**Step-by-Step Instructions for Finding NHANES Datasets**

**Step 1: Visit the NHANES Website**

1. Go to the NHANES official website: https://www.cdc.gov/nchs/nhanes/index.htm.
2. On the homepage, find and click "*Data & Documentation*" to enter the data access section.

**Step 2: Choose a Survey Cycle**

1. NHANES data are released in two-year cycles. Each cycle provides a snapshot of the U.S. population for that period.
2. The available cycles are listed, ranging from 1999-2000 to the most recent 2019-2020. For example: If your study involves data collected between 2017 and 2020, select these cycles for your dataset.
3. Click the specific cycle link to view the data files.

**Step 3: Select the Relevant Data Category**

NHANES organizes datasets into the following categories. Choose files based on your study needs:

1. Demographics Data: Includes information on age, gender, ethnicity, education, income, and marital status. Click on "*Demographics Data*" and download the **.XPT** file corresponding to the cycle.
2. Examination Data: Contains results from physical exams, imaging, or diagnostic tests (e.g., body measurements, liver ultrasound results). Navigate to "*Examination Data*" and identify relevant data files.
3. Laboratory Data: Includes biochemical and clinical test results (e.g., serum uric acid, cholesterol levels, glucose levels). Go to "*Laboratory Data*" and download the required files for your study.
4. Questionnaire Data: Includes self-reported health information, such as conditions like gallstones (GS), dietary intake, or physical activity. Navigate to "*Questionnaire Data*" and locate the questionnaire on gallstone history or dietary recalls.

**Step 4: Access Documentation (Codebooks)**

1. Every dataset includes a Codebook to describe variables, values, and units.
2. Download the Codebook (available as a PDF or TXT file) to understand: Variable names (e.g., MCQ160L for gallstone diagnosis). Value coding (e.g., "1" = Yes, "2" = No).
3. Use the Codebook to interpret your dataset correctly.

**Step 5: Download Data Files**

1. The data files are provided in **.XPT** format, compatible with statistical software like R, Python, SAS, or Stata.
2. Click "*Data*" next to each file and download it to your computer.
3. Save the files in an organized directory for further analysis.

**Step 6: Open and Process the Data**

1. Open the **.XPT** files in software (R, Python, SAS or Stata)
2. Merge datasets using the unique participant identifier variable SEQN to combine demographic, laboratory, examination, and questionnaire data.

**Step 7: Analyze the Data**

1. Clean and prepare the data according to your study design.
2. Perform statistical analyses, such as logistic regression or subgroup analysis, to test your hypotheses.

**Additional Tips**

1. Search Keywords: Use relevant keywords to locate datasets in the NHANES database, such as “serum uric acid,” “gallstones,” or “dietary recall.”
2. Tutorials: NHANES provides tutorials on data usage and merging files. Access these at: https://wwwn.cdc.gov/nchs/nhanes/tutorials.aspx.
3. Ethical Considerations: NHANES data are de-identified and publicly available, but always cite the source appropriately.
